# Finding needles in a haystack: identification of inter-specific introgressions in wheat genebank collections using low-coverage sequencing data

**DOI:** 10.3389/fpls.2023.1166854

**Published:** 2023-06-06

**Authors:** Jens Keilwagen, Heike Lehnert, Ekaterina D. Badaeva, Hakan Özkan, Shivali Sharma, Peter Civáň, Benjamin Kilian

**Affiliations:** ^1^ Institute for Biosafety in Plant Biotechnology, Julius Kuehn Institute, Quedlinburg, Germany; ^2^ N.I. Vavilov Institute of General Genetics, Russian Academy of Sciences, Moscow, Russia; ^3^ The Federal Research Center Institute of Cytology and Genetics, Siberian Branch of the Russian Academy of Sciences (ICG SB RAS), Novosibirsk, Russia; ^4^ Department of Field Crops, Faculty of Agriculture, University of Çukurova, Adana, Türkiye; ^5^ Global Crop Diversity Trust, Bonn, Germany; ^6^ Université Clermont Auvergne, INRAE, Génétique, Diversité et Ecophysiologie des Céréales, Clermont-Ferrand, France

**Keywords:** plant genetic resources, crop wild relatives, genetic diversity, interspecific introgression, coverage, duplicates, heterogeneity, *Triticum*

## Abstract

Recently, entire genebank collections of wheat have been extensively characterized with sequencing data. We have identified introgressions using these genotyping-by-sequencing and whole-genome sequencing data. On the basis of our results, we provide information about predicted introgressions at 1-Mb resolution for 9,172 wheat samples as a resource for breeders and scientists. We recommend that all plant genetic resources, including genebank collections, be characterized using a combination of variant calling and introgression prediction. This is necessary to identify potential duplicates in collections efficiently and reliably, and to select promising germplasms with potentially beneficial introgressions for further characterization and prospective breeding application.

## 1 Introduction

The recognition of the importance of plant genetic diversity for crop improvement and the loss of agrobiodiversity (also refer to as genetic erosion) led to the establishment of the first seedbanks in the early 20^th^ century (Scarascia-Mugnozza and Perrino, 2002; Gepts, 2006; Díez et al., 2018; Salgotra and Chauhan, 2023). Today, more than 7.5 million samples of plant genetic resources for food and agriculture (PGRFA) are held in approximately 1,800 seedbanks (also refer to as genebanks) (Knüpffer, 2009; Weise et al., 2020; Shaw et al., 2023). These genebanks are ultimate repository of native beneficial diversity. The impact of climate change and other challenges can be better addressed by sufficient crop diversity in the breeding pools and the entire global food system (Cortés, 2017; Razgour et al., 2019; Sharma et al., 2021). Overall, genebanks play a pivotal role in meeting the United Nations Sustainable Development Goal 2 “End hunger, achieve food security and improve nutrition and promote sustainable agriculture” (Johnson, 2008) (https://unstats.un.org/sdgs/report/2016/goal-02/).

For many reasons, including missing genotypic and phenotypic data, most genebank collections have yet to be used for crop improvement (Anglin et al., 2018; Pathirana and Carimi, 2022). However, landraces and crop wild relatives (CWR) harbor desired traits, for example, resistance or tolerance to biotic or abiotic stresses (Bhullar et al., 2010; He et al., 2019; Cseh et al., 2021; Bohra et al., 2022; Leigh et al., 2022; Salgotra and Chauhan, 2023). These traits can be transfered into crop plants via intra- or interspecific hybridization (Wulff and Moscou, 2014; Molnár-Láng et al., 2015; Dempewolf et al., 2017; Hao et al., 2020; Ayed et al., 2021; Kilian et al., 2021; Singh et al., 2021; Eastwood et al., 2022). Hybridization can either occur as a natural process or as a deliberate and controlled process during breeding. During hybridization, DNA from a donor is integrated into the genome of a crop plant, and these newly transfered DNA sequences are known as introgressions. Introgressions can lead, for example, to substitutions or presence/absence variations. Interspecific hybridizations are often challenging due to crossability barriers (Khush and Brar, 1992; Singh et al., 2021; Laugerotte et al., 2022). Hence, finding and using existing introgressions in genebanks might accelerate crop plant improvement.

Advances in next-generation sequencing (NGS) technologies enable the genotypic characterization of extensive germplasm collections. Genebanks have started using genomics tools and will take advantage of the wealth of genomic data that are being produced (Kilian and Graner, 2012; Wambugu et al., 2018; Wambugu and Henry, 2022). These genomic data make it possible to quantify variation within and between accessions (van Treuren and van Hintum, 2003; Mace et al., 2006; Upadhyay et al., 2013; Singh et al., 2019), and will play an essential role in increasing the efficiency of genebank management by allowing managers to make informed decisions about reducing redundancy in germplasm collections. Further, precise evaluation of heterogeneous germplasm accessions for the traits of breeders’ interest requires minimizing within-accession variability, so single seed descent (SSD) lines are frequently produced and genotyped (Brown, 1989; Anglin et al., 2018; Singh et al., 2019; Kroc et al., 2021; Rocchetti et al., 2022).

Several different genotyping protocols have been developed, e.g., genotyping-by-sequencing (GBS), which reduces genome complexity by sequencing only specific genomic fragments (Poland and Rife, 2012). Alternatively, whole genome sequencing (WGS) provides a more detailed view of the genome. The rapid decrease in sequencing costs means that it is now possible to genotypically characterize whole genebank collections (Milner et al., 2019; König et al., 2020; Sansaloni et al., 2020). Sequencing data are mainly used for variant calling, and downstream analysis steps rely on these variants.

Recently, Schulthess et al. (2022a) used both GBS and WGS for the genetic profiling of an extensive winter wheat genebank collection comprising genebank accessions, elite cultivars, and elite inbred lines from a breeder’s panel to identify potential donors of resistance. Keilwagen et al. (2022) detected major introgressions in the genome assemblies of several wheat cultivars and the putative donor species using short-read data. This method can be adapted to detect introgressions in diverse collections if a reference genome sequence and short read data are available.

Here, we reanalyzed the data of Schulthess et al. (2022a), aiming to identify introgressions that potentially harbor beneficial traits for wheat breeding.

## 2 Materials and methods

### 2.1 Material

The German Federal *ex situ* genebank at IPK Gatersleben harbors one of the largest wheat collections worldwide (https://www.ipk-gatersleben.de/en/infrastructure/gene-bank). Recently, Schulthess et al. (2022a) conducted (i) GBS for 7,651 genebank accession numbers and 325 elite wheat cultivars; and (ii) WGS for 444 genebank accessions and 322 elite cultivars and advanced breeding lines.

In some cases, different phenotypes were observed for a specific accessions. Hence, those accessions were represented by more than one SSD line.

In total, 94 heterogeneous genebank accessions represented by two SSD lines and 171 cultivars represented by three independent samples were genotyped with GBS. For WGS, only two genebank accessions were genotyped using two independent SSD lines. All the remaining samples were genotyped once.

In total, GBS and WGS experiments were run for 8,412 and 768 samples, respectively. GBS and WGS data of the same genebank accessions can be identified by the SSD-PGR, while for cultivars the cultivar name can be used.

Samples genotyped with WGS were assigned to three groups by (Schulthess et al 2022a; Schulthess et al 2022b): (i) the “Pre-Green Revolution”, consisting of landraces and cultivars released before 1970; (ii) the “Old Cultivar Panel”, consisting of cultivars released between 1971 and 2000; and (iii) the “New Cultivar Panel”, consisting of cultivars released after 2000.

Here, these data were reanalyzed with an aim to identify large introgressions. All analyzed data are publicly available at the European Nucleotide Archive (https://www.ebi.ac.uk/ena/) under the project IDs PRJEB41976, PRJEB4873, and PRJEB48988.

### 2.2 Methods

Coverage analysis was adapted from Keilwagen et al. (2022) and used for the GBS and WGS data. For GBS, the complete dataset was used, while down-sampled data were used for WGS. Raw sequencing data were adapter- and quality-trimmed with Trim Galore (version 0.4.0; non-default parameters: quality ≥ 30, read length ≥ 50; https://github.com/FelixKrueger/TrimGalore). Trimmed reads were individually mapped against the wheat reference genome of Chinese Spring v2.1 (Zhu et al., 2021) using BWA-mem (v0.7.15-r1140) (Li, 2013). A concatenated reference sequence of Chinese Spring version 2.1 and rye (Rabanus-Wallace et al., 2021) was used to infer the potential origin of introgressions. Unmapped reads, supplementary reads, and non-primary alignments were removed from mapped reads using SAMtools (version 1.10.2; –F 2308) before computing sequencing depth (Li et al., 2009). The percentage of bases covered 1-Mb window was computed with custom Java and R scripts.

Introgressions were identified with R (R Core Team, 2022) using a four-step approach for WGS and GBS data. Only the first step slightly differed between GBS and WGS data analyses due to the use of methylation-sensitive restriction enzymes in GBS and different methylation patterns in telomeric and centromeric regions.

First, initial calling was performed using a threshold-based method. For WGS, a window was initially called if the absolute difference between the overall median and the window’s value was larger than 1.5 times the median absolute deviation. Depending on the sign of the difference between the overall median and the value of the window, the labels 1 and -1 were given. Not-called windows were labeled with 0. For GBS data, the initial calling used the percentage of bases covered in a GBS experiment of the reference Chinese Spring (SAMEA5374255). The values of the reference and sample were normalized by their own median percentage of bases covered. The relative deviation was computed when comparing the values of the sample investigated with those of the reference, Chinese Spring. If the absolute value of relative deviation was more than 20%, the window was initially called using the sign of the relative deviation as a label.

Second, only stretches of at least five initial calls in a row with the same label were used in further analyses. Smaller stretches were deleted (=set to 0) for denoising purposes.

Third, gaps between neighboring calls from step two with the same label also received this label if the signs of all the corresponding values of step one equaled the label of the neighboring calls.

Fourth, stretches with fewer than 25 identically labeled windows in a row were deleted (set to 0). Hence, small introgressions were ignored in this analysis and only introgressions with a size of at least 25 Mb made it to the final prediction. Visualization was done with R.

Fisher’s exact test was used to determine regions with increased number of introgression predictions from GBS in the group of elite cultivars compared to the group of genebank accessions.

## 3 Results

Introgressions of at least 25 Mb size were identified based on the percentage of bases covered along the Chinese Spring version 2.1 genome assembly. Information about these identified introgressions is available as a resource for breeders and scientists. The introgressions are shown in 1-Mb resolution (Keilwagen, 2023) in e!DAL (Arend et al., 2014) and as a summary (**Supplementary Tables 1**, **2**).

### 3.1 Overview of introgressions detected

Based on the low percentage of bases covered in one wheat genome, 22 samples from the GBS data were marked with the tag “missing genome” (**Supplementary Table 1**). In these 22 samples, the D genome had low coverage. These samples perfectly matched those identified and confirmed as not hexaploid wheat samples by Schulthess et al. (2022a).

**Figure 1:**
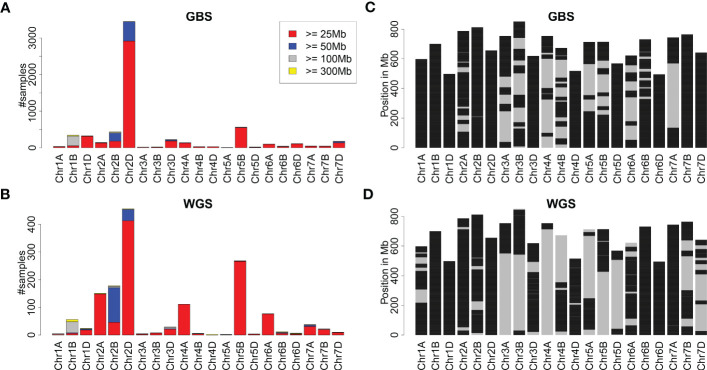
Overview of introgressions in wheat collections genotyped with GBS and WGS. **(A, B)** depict the number of samples with introgressions of at least 25, 50, 100, and 300 Mb per chromosome as identified from GBS and WGS data. **(C, D)** depict the genomic locations of wheat chromosomes covered by any introgression prediction in black. Due to the different collections and their sizes, **(C, D)** are not directly comparable.

The remaining samples were treated as hexaploid wheat. An overview of all introgressions identified is depicted in **Figure 1**. Most introgressions identified from both the GBS and WGS data were moderate in size (25-50 Mb). Nevertheless, about 78% and 66% of the wheat genome was covered by any introgression prediction using GBS and WGS data, respectively. For some of the chromosomes, each genomic window was covered by any introgression (**Figure 1**). The identified introgressions were clustered in certain genomic regions (**Supplementary Figure S1**). The region with the most samples harboring an introgression of at least 25 Mb was chromosome 2DL. Other prominent chromosomal regions harboring introgressions were 1BS, 1DL, 2BL, and 5BL as detected from the GBS data and 1BS, 2AS, 2BL, 4AL, 5BL, and 6AS as detected from the WGS data.

Comparing introgression predictions based on GBS data, 12 regions were identified where introgressions were predicted more often in elite cultivars compared to genebank accessions (**Supplementary Table 3**). Nine out of these 12 regions overlap with regions identified by Schulthess et al. (2022a) using WGS data, while the regions on chromosomes 3DS, 6DS and 7DS were not described. Interestingly, seven out of 12 regions are located on the D subgenome.

Comparing the frequency of introgressions for GBS and WGS, apparent differences were observed for some chromosomes, including 2A, 2B, 4A, 5B, and 6A (**Figure 1**). These differences in the observed frequencies of introgressions might be attributed, for instance, to the different collections of genotypes analyzed and to the experiment type, namely GBS and WGS. Comparing the occurrence of introgressions per chromosome and historical period in the WGS collection, an enrichment of introgressions on chromosomes 2A, 2B, 2D, and 5B was visible for the New Cultivar Panel (**Supplementary Figure S2**), indicating a selection bias due to the higher number of more recently released cultivars in the WGS collection. In contrast, the number of samples with introgressions on chromosome 4A changed only slightly over the three historical periods. Still, introgressions on chromosome 4A were often identified in the collection genotyped with WGS, indicating that these introgressions could be identified more easily with WGS than with GBS. Fewer samples with introgressions on chromosome 6A were present in the New Cultivar Panel than in the Old Cultivar Panel and the Pre-Green Revolution Panel.

**Figure 2:**
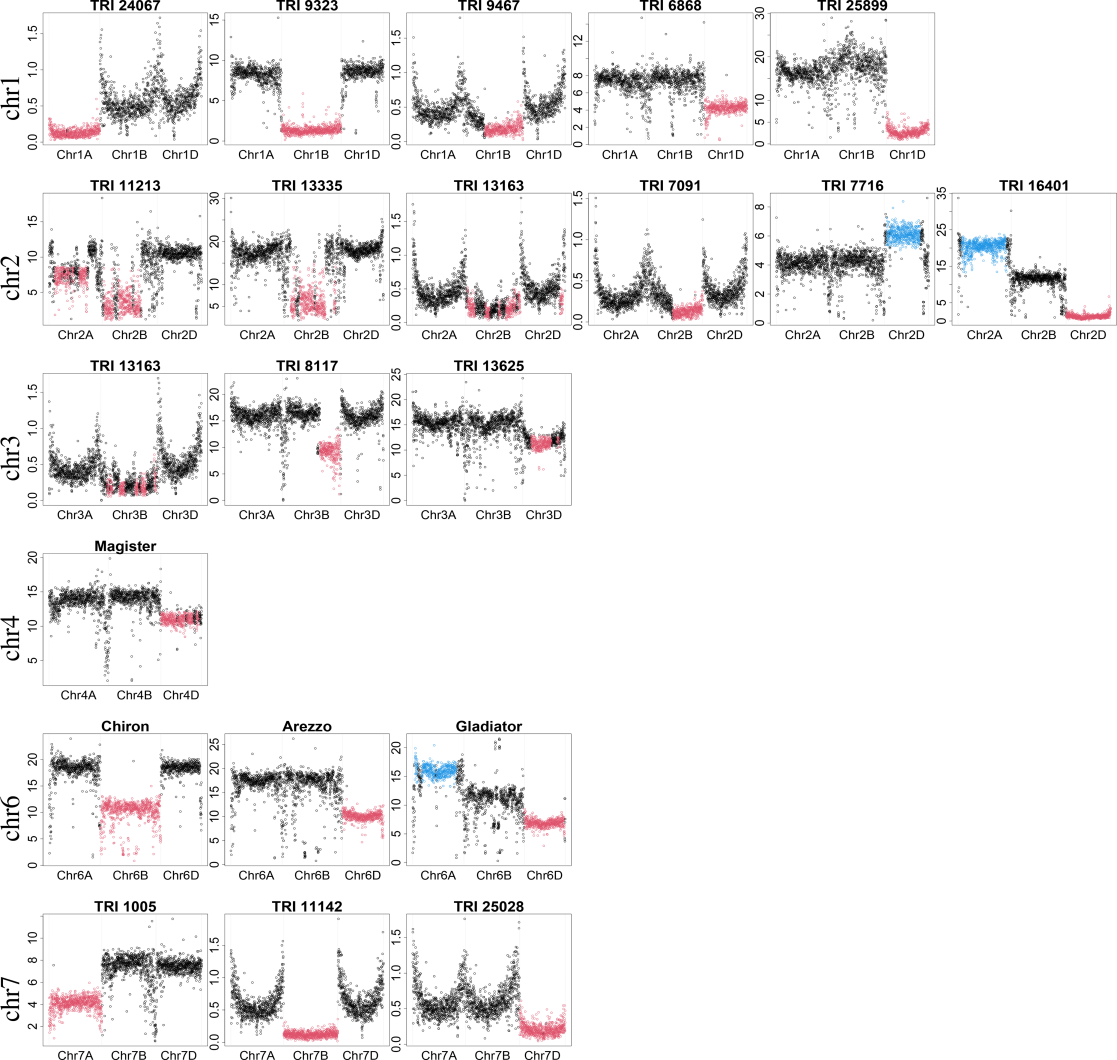
Examples of detected large introgressions (≥ 300 Mb). Dots show the percentage of bases covered in 1-Mb windows. X-axis indicates chromosomal location; scale on y-axis depicts the percentage of bases covered and depends on the experiment (GBS or WGS) and sequencing depth. Absolute values are not of interest, but rather the ratio between them. Consistent with introgression detection, black color indicates regions with expected percentage of bases covered. Red and blue colors indicate regions with an unexpectedly low and high percentage of bases covered, respectively.

Despite being very conservative, focusing only on introgressions of at least 25 Mb, 130 samples were identified to have introgressions in the WGS dataset in this analysis, but were determined to have no introgressions in the original study by Schulthess et al. (2022a). Most of these introgressions had a moderate size (slightly larger than 25 Mb). However, some samples with large introgressions were also identified, e.g., TRI 1005 with an introgression on chromosome 7A and TRI 7716 with an introgression on chromosome 2D (**Figure 2**; **Supplementary Table 2**).

Although each individual identified introgression might be valuable, and especially frequently occurring introgressions might confer desirable traits, we focused on the large introgressions. These large introgressions might be known and already used for breeding, such as 1R/1B, or unknown and yet to be characterized. Identified introgressions harboring beneficial alleles might be a starting point for increasing wheat diversity in breeding pools. Nevertheless, all identified introgressions, including smaller ones, are provided at 1-Mb resolution to the wheat research community for further analyses (Keilwagen, 2023).

### 3.2 Scrutinizing large introgressions (≥ 300 Mb)

**Supplementary Table 4** lists 81 samples harboring at least one chromosome with a large introgression (≥300 Mb) identified from either GBS or WGS data. Large introgressions were found on different chromosomes, comprising one sample for 1A, 31 samples for 1B, four samples for 1D, four samples for 2A, 16 samples for 2B, eight samples for 2D, two samples for 3B, two samples for 3D, two samples for 4D, one sample for 6A, five samples for 6B, three samples for 6D, one sample for 7A, three samples for 7B, and four samples for 7D.

Some prominent representatives of these samples are depicted in **Figure 2**. For each representative, the three homoeologous chromosomes are shown. For some samples, e.g., TRI 11213 and TRI 16401, introgressions were identified on more than one chromosome. This could be either multiple true introgressions or substitution of a genomic region by a region of a donor species more similar to another wheat chromosome. In this case, the percentage of bases covered in a genomic region is likely lower than expected, while the percentage of bases covered will be higher than expected for the other region. Sequence data for all large introgressions were mapped against the concatenated reference wheat and rye genome assembly to determine if the donor of the introgression was rye.

An introgression on chromosome 1A was identified in TRI 24067 (Kadolzer St 3), whereas introgressions on chromosome 1B were identified in many samples. Comparisons with the concatenated reference revealed that substitution of chromosome 1B by chromosome 1R from rye, termed 1B/1R, was present in 28 samples, including Feldmann, Mildress, Salzmünder 14/44, Sholeh, and Zorba (Schlegel and Korzun, 1997). In addition, different introgression profiles from rye to 1B were detected in two samples (TRI 25875, TRI 24963) (**Supplementary Figure S3**). A smaller introgression was identified in TRI 9467 (ns 739). Comparison with the concatenated reference indicated that this introgression originated from 1RL and is designated as 1BS.1RL. Several samples with 1RS.1BL were also detected but those introgressions were smaller than the threshold of 300 Mb. Large introgressions on chromosome 1D were identified in TRI 6868 (Roux De Champigny) and TRI 25899. The ratio of the median percentage of bases covered for chromosome 1D compared with 1A was 0.57 (4.30 vs. 7.51) for TRI 6868 and 0.17 (2.69 vs. 16.26) for TRI 25899. These ratios indicated differences in the similarity of the introgressed region to the original chromosome 1D and, hence, different donors. In addition, TRI 25875 was identified to have a smaller introgression on chromosome 1DS.

Two large introgressions on chromosome 2A and 2B were identified in TRI 11213, while an introgression on chromosome 2B was identified in many samples, e.g., TRI 13335 (Hand) and TRI 13163. For many but not all of those samples, an introgression from *Triticum timopheevii* was identified based on pedigree analysis (Keilwagen et al., 2022). A smaller introgression on chromosome 2B was identified in TRI 7091 (Honor (2) - Rosen rye X Yorkwin - Cornell 595). Comparison of TRI 7091’s sequence data against the concatenated reference sequence revealed an introgression on 2RL, which has been described for crosses with Rosen Rye (Crespo-Herrera et al., 2017). An introgression on chromosome 2D was identified in TRI 7716 (Iva) with an unexpectedly high percentage of bases covered. Another interesting case was TRI 16401 (Duck), which had a low percentage of bases covered on chromosome 2D, in contrast to a high percentage of bases covered on chromosome 2A, suggesting substitution of 2D by a 2A-like chromosome.

Multiple introgressions were identified in TRI 13163, including an introgression on chromosome 3B besides 1RS.1BL and an introgression on chromosome 2B. An introgression on chromosome 3BL was identified in TRI 8117 (Xi-Bei 612), and one on chromosome 3D was identified in TRI 13625. In addition, we detected an introgression on chromosome 3D in TRI 24699 (Timpaw) that is known to originate from *Thinopyrum ponticum* (syn. *Agropyron elongatum*) and contains the resistance genes *Lr24/Sr24* (McIntosh et al., 1995).

Of chromosomes 4A, 4B, and 4D, only 4D was identified to harbor large introgressions. An introgression was identified on chromosome 4D for Magister and also for Rywalka.

An introgression on chromosome 6B was identified in Chiron and TRI 17920. In addition, a smaller introgression on chromosome 6BS was identified in TRI 22103, TRI 5164 (Cappelle Desprez), and TRI 9447 (D 130/63). An introgression on chromosome 6D was identified in Arezzo. Another interesting case was the elite wheat cultivar Gladiator, which had a low percentage of bases covered on chromosome 6D, in contrast to a high percentage of bases covered on chromosome 6A, suggesting substitution of 6D by a 6A-like chromosome. A similar case was found for the old landrace TRI 259 (Mahndorfer Tempo).

An introgression on chromosome 7A was identified in TRI 1005 (Algebra), and one on chromosome 7B was identified in TRI 11142. A similar pattern was observed in TRI 25137 (Purdue 39120 A4-2-2-2-2-2). A smaller introgression on chromosome 7BS was detected in TRI 13645. An introgression on chromosome 7D was identified in TRI 25028 (Agrus). In Agrus, chromosome 7D has been substituted by 7Ag from *Thinopyrum ponticum*, which harbors the resistance gene *Lr19* (McIntosh et al., 1995; Šliková et al., 2003). Similar patterns on chromosome 7D were found in another Agrus accession (TRI 8300), as well as B 96 (Purdue 39120) and B 97 (Purdue 5392).

To the best of our knowledge, many of the large introgressions identified here were previously unknown, especially if samples were unnamed, e.g., TRI 13625 and TRI 17920, or old cultivars or landraces, e.g., TRI 6868 (Roux De Champigny) and TRI 24067 (Kadolzer St 3). On some chromosomes, including 5A, 5B, and 5D, no large introgressions (≥ 300 Mb) were found, but somewhat smaller introgressions were identified (**Supplementary Tables 1**, **2**). Interestingly, large introgressions were found on all chromosomes of the D genome of wheat except 5D, offering potential beneficial diversity that can be exploited for crop improvement.

### 3.3 Duplicates and heterogeneity

Genebank accessions might not consist of a single genotype, especially if they are landraces or CWR (Keilwagen et al., 2014; Singh et al., 2019; Mihelich et al., 2020; Badaeva et al., 2021). Some genebanks, including IPK, have established protocols for splitting accessions based on phenotypic differences in characterization and regeneration trials (Hintum et al., 2002). However, accessions may still be mixtures of different genotypes after line splitting. Based on phenotypic differences detected in specific genebank accessions, Schulthess et al. (2022a) genotyped more than one SSD line per accessions. Hence, some accession or cultivar names occurred more than once in the datasets for various reasons. Some of these samples belonged to the same genebank accessions, but two SSD lines were produced and analyzed per accessions (SSD-PGR), e.g., TRI 3810. Others had different accession numbers but similar or identical accession names, e.g., TRI 8018 (Riebesel ST. 47-51) and TRI 24963 (Riebesel 47/51). In both cases, the profile the percentage of bases covered on chromosome 1B differed, indicating different genotypes, possibly due to different introgressions (**Supplementary Figure S3**).

Additionally, four extreme examples of accessions with two genotyped SSD lines and at least 100- Mb differences between them on one chromosome were detected from the GBS data (see **Supplementary Figure S4**). The accession TRI 7878 was a mixture of a tetraploid and hexaploid wheat. Accession TRI 16401 (Duck) had one SSD line with a substitution of 2D by a 2A-like chromosome. The accession TRI 10859 had one SSD line with 1RS.1BL. The accession TRI 7025 (Red rock) had one SSD line with an introgression on 2BS. These examples show that genebank accessions can be mixtures of several genotypes and that different introgressions can be detected within some genebank accessions.

However, other examples with similar accession names or the same accession number showed very similar profiles.

### 3.4 Comparison of different low-coverage data

Finally, introgression predictions based on WGS and GBS data were directly compared. To reduce the number of examples, only samples with at least one large introgression were considered (23 samples). Different levels of homogeneity can be expected for genebank accessions and cultivars, so the samples were divided into genebank accessions and cultivars. Each recently released cultivar should be a single genotype (genetically homogeneous or identical), as seed purity is required for the release process. At the same time, genebank accessions, which can be landraces and old cultivars, can be a mixture of several genotypes, as described above. Therefore, genebank accessions were distinguished as accessions with the same descent (SSD-PGR) and accessions with unknown descent.

Six accessions with unknown descent and at least one large introgression were found, and significant differences were found in four of these six accessions (**Supplementary Figure S5**). Large introgressions or substitutions were identified on the complete chromosome 7A in accession TRI 1005, on the short arm of chromosome 6B in accession TRI 5164, on the full chromosome 1D in accession TRI 6868, and on almost the complete chromosome 2D in accession TRI 7716.

Eleven accessions with the same descent and at least one large introgression were found, and large differences were found in four of them (**Supplementary Figure S6**). In one of these four cases (TRI 11213), it was challenging to identify the introgressions on chromosome 2A and 2B from the GBS data. Another case was TRI 24963, where a different rye introgression was identified from the WGS data but not the GBS data (**Supplementary Figure S3**). Because rye introgressions and substitutions on chromosome 1B could be detected from the GBS data (**Supplementary Figure S3**), this sample could be a mixture, despite having the same descent/SSD-PGR. In the remaining two cases, there were significant differences in introgressions identified based on GBS and WGS data despite the same descent/SSD-PGR. An introgression was identified on the long arm of chromosome 3B in TRI 8117 and one on chromosome 3D was identified in TRI 13625.

In addition, six recently released cultivars with at least one large introgression were found (**Supplementary Figure S7**). Schamane had a 1R/1B substitution according to the WGS data, whereas a normal chromosome 1B was indicated by the GBS data. Substitution of wheat chromosome 1B by rye chromosome 1R was typically detected from the GBS data (**Supplementary Figure S3**). Thus, it could be a case of confounding. In the remaining five cultivars, introgressions were located on chromosome 6D in Arezzo, on chromosome 6B in Chiron, on chromosome 6A and 6D in Gladiator, and on chromosome 4D in Magister and Rywalka.

In summary, 11 samples showed differences in detected introgressions between GBS and WGS data (four accessions with unknown SSD-PGR, two with the same SSD-PGR, and five cultivars). In all cases, the differences were related to predicted introgressions based only on WGS data but not GBS data. Often, the difference between the expected percentage of bases covered in the wheat chromosome and the observed percentage of bases covered in the introgression was visible but smaller compared with other introgressions.

LG Magirus was another example where an introgression was detected from the WGS data but not the GBS data. This was because of the small difference between the expected and observed percentage of bases covered. The introgression was located on chromosome 1B. Still, the size did not exceed the threshold of 300 Mb due to the similarity between the introgressed donor sequence and the replaced sequence of chromosome 1B (**Supplementary Figure S3**).

Since WGS data are better suited for identifying introgressions than are GBS data, and WGS is much more expensive than GBS, the question arises as to what sequencing depth is needed to predict introgressions from WGS data. Hence, we used publicly available WGS data for cultivars that harbor large introgressions and varied the sequencing depth, i.e., the number of raw reads per sample, from 0.5 to 25 million reads. Three cultivars were selected to cover different introgression donors, and introgressions were predicted independently for each sequencing depth (**Supplementary Figure S8**). Introgressions could be detected for the first time at different sequencing depths; e.g., the introgression of rye in Schamane was detected even with 0.5 million raw reads. In contrast, the introgression in Magister was detected only with 25 million raw reads. This corresponds to 0.005- to 0.26-fold coverage of the wheat genome.

## 4 Discussion

The rapid decrease in sequencing costs has allowed for genotypically of extensive genebank collections using sequencing protocols like GBS and WGS. Recently, Schulthess et al. (2022a) genotypicly characterized an extensive winter wheat collection containing genebank accessions and breeding lines, and screened for resistance genes. In addition, introgressions ranging in size from 19.6 to 50 Mb were detected on chromosomes 1B, 1D, 2A, 2B, 2D, 3D, 4A, 5B, and 7D from the WGS data.

Based on these data and an adapted method (Keilwagen et al., 2022), introgressions with a minimum size of 25 Mb were detected in the present study on all chromosomes using the GBS and WGS data. Some of these chromosomal regions harboring introgressions are well known. The most widespread introgressions are on chromosome 1B. These are a substitution of chromosome 1B by rye chromosome 1R, referred to as 1R/1B, and translocations of rye chromosome arm 1RS onto the wheat chromosome arm 1BS, referred to as the 1BL.1RS translocation. More than 1000 wheat lines and cultivars carrying the 1BL.1RS translocation have been developed worldwide (Rabinovich, 1998; Jiang et al., 2017; Schlegel, 2022). Wheat cultivars carrying rye introgressions are being extensively grown on over five million hectares worldwide (Villareal et al., 1991; Kumar et al., 2003). Further known introgressions include those on chromosome 2AS from *Aegilops ventricosa* (Bariana and McIntosh, 1993; Helguera et al., 2003; Gao et al., 2021) and chromosome 2B from *Triticum timopheevii* (Friebe et al., 1996; Walkowiak et al., 2020; Keilwagen et al., 2022). In addition, introgressions on chromosome arm 2DL (Thind et al., 2018; Keilwagen et al., 2019; Keilwagen et al., 2022) and chromosome 5B (Keilwagen et al., 2019; Schulthess et al., 2022a) have been reported, but their origin is either not known or not yet fully resolved.

Searching for regions with enriched introgression frequency in elite cultivars compared to genebank accessions, 12 regions have been identified using GBS data. These regions overlap well with previously described regions (Schulthess et al., 2022a), but also additional regions were identified which need to be validated in the future.

Large introgressions (at least 300 Mb per chromosome) were identified on 15 chromosomes, including 1A, 3B, 4D, 6A, 6B, 6D, 7A, and 7B, which were not reported to have introgressions by Schulthess et al. (2022a). Some of these large introgressions are known from the breeding history, e.g., 1R/1B in Salzmunder 14/44 (Rabinovich, 1998) and 7Ag/7D in Agrus (McIntosh et al., 1995; Šliková et al., 2003). In contrast to these known introgressions, many others were, to the best of our knowledge, unknown until now. In conclusion, we have shown for the first time that introgressions in large wheat genebank collections can be successfully predicted using GBS or ultra-low coverage (ulc) WGS (≤ 0.5X) data. It is not meaningful to compare the absolute cost per sample for GBS and ulcWGS because it depends on many factors, e.g., the service provider and the total amount of samples, and becomes outdated very quickly. However, 25 million raw data per sample for ulcWGS is still much more than the average for GBS in wheat and therefore GBS is cheaper.

These results show that plant genetic resources conserved in genebanks harbor a wealth of introgressions, most of which are yet to be characterized and utilized. These introgressions should be easier to use in breeding programs because they are already present in domesticated materials (landraces or elite cultivars). Ultimately, these unknown or underutilized introgressions should be examined for their potential uses in wheat improvement; e.g., Kadolzer stamm 3 (TRI 24067) carries an introgression on chromosome 1A and has been described as drought tolerant (Rademacher, 1947). Whether its drought tolerance is related to this introgression is yet to be investigated. Some accessions carrying 1RS and 7Ag are also described as drought tolerant. However, these introgressions were originally utilized for a different purpose (Ehdaie et al., 2003; Placido et al., 2013). We are unaware of reported phenotypes for other samples with large introgressions. Detailed phenotyping of accessions with large introgressions could uncover novel beneficial loci or alleles. In addition, several accessions with introgressions in the D genome were identified, and these could increase diversity in the D genome for use in breeding programs (Mirzaghaderi and Mason, 2019). All identified introgressions at 1-Mb resolution for each sample will now be available as a resource for breeders and scientists.

Directly comparing introgressions identified from GBS and ulcWGS data for genebank accessions and cultivars, 11 samples with large differences were identified. In all 11 cases, the ulcWGS data led to the discovery of additional introgressions. The difference between the expected and observed percentage of bases covered was visible for these introgressions but was smaller than for other introgressions. This indicated that those introgressions were more similar to the original DNA fragment. With ulcWGS, we could detect these minor differences in the percentage of bases covered because the whole genome is considered, not just a small portion, and no (methylation-sensitive) restriction enzymes are used. Our results show that the detection sensitivity depends not only on the sequence data type, sequencing depth, and introgression donor, but also on the introgression size and detection method.

Alignment of short sequence reads from a genebank accession with an introgression to the reference genome sequence without this introgression reveals an unexpectedly high or low percentage of covered bases, which was used to predict introgressions. These profiles of percentage bases covered indicate that introgressions could result in increased or decreased coverage on some chromosomes, with decreased coverage reflecting the absence of the expected region. In contrast, increased coverage may indicate that an introgression (of another region) is more similar to this genomic region. Variant calling using a reference genome sequence is based on mapped reads and could be problematic in such regions, leading to either heterozygous or incorrect variant calls or an increased number of missing values. For this reason, increased or decreased coverage may impact variant calling and all downstream analyses, including imputation, population genetics, and genome-wide association studies.

Differences in introgressions identified in different plants with the same genebank accession number confirm that genebank accessions can be heterogeneous (Keilwagen et al., 2014; Keilwagen et al., 2022). However, Schulthess et al. (2022a) only analyzed two independent SSD lines for accessions with a clear phenotypic difference. Hence, we cannot give an unbiased prediction for the percentage of heterogeneous genebank accessions based on the introgressions identified here. The generation of SSD lines brings advantages for further analysis but may lead to an underestimation of genetic diversity in genebank accessions and, thus, whole genebank collections.

For the same reasons, the detection of duplicates in genebank collections must be reconsidered. In general, duplicates are a problem for genebanks due to the enormous amount of time, labor, and money spent on identical genotypes that do not contribute to research or breeding (Dobrovolskaya et al., 2005; Singh et al., 2019; Pathirana and Carimi, 2022). Hence, eliminating duplicates is a high priority for genebanks. However, some genebank accessions do not have accession names, passport data, or pedigree information that might be used to detect duplicates. Even for cultivars like Riebesel st. 47-51, neither the accession number nor the accession name can be used to identify duplicates, as demonstrated here. For these reasons, computational methods based on single-nucleotide polymorphisms (SNPs) and genetic distances have been proposed (Singh et al., 2019; Cseh et al., 2021; Sahu et al., 2022; Schulthess et al., 2022a). Still, these methods might have problems with introgressions leading to missing variant calls due to low coverage in the corresponding genomic regions as described above. For instance, Schulthess et al. (2022a) used SNP data and clustered accessions based on their proportion of pairwise difference. Using this method, they defined, for instance, cluster C2 with 766 identical genotypes. Based on our introgression analyses, this cluster contained several samples with medium-size introgressions of at least 100 Mb, comprising 12 samples with large introgressions on 1B and one with a large introgression on each of chromosomes 2A, 2B, 2D, 5A, and 5D (**Supplementary Table 1**). These samples cannot be duplicates, and removing them from genebank collections might cause a loss of genetic diversity and potentially beneficial alleles.

Hence, duplicate detection should not be based on a single SSD line per accession number. In addition, a pure SNP-based approach might miss introgressions, which might be particularly important for pre-breeding and breeding programs. For these reasons, multiple randomly selected seeds/plants from each existing accession number and each new genebank entry should be analyzed individually using a combination of introgression identification and variant calling to detect potential duplicates. The number of analyzed plants per accession will determine the detection threshold for the fraction of this accession that might be a different genotype. UlcWGS is a reasonable approach allowing for variant calling (Chat et al., 2022) and introgression prediction. Duplicates could be detected based on a two-step approach. First, introgressions should be predicted; and second, variant calling could be used for samples with similar predicted introgressions to detect duplicates. Finally, genebank documentation systems (e.g., https://www.agent-project.eu/, https://www.pulsesincrease.eu/) should provide access to all data including introgression and variant data to efficiently identify promising materials for more detailed evaluation and potential application in targeted crop improvement.

## Data availability statement

Publicly available datasets were analyzed in this study. The datasets analyzed for this study can be found in the European Nucleotide Archive (ENA): https://www.ebi.ac.uk/ena/browser/view/PRJEB41976 (GBS genebank collection), https://www.ebi.ac.uk/ena/browser/view/PRJEB48738 (WGS genebank collection), https://www.ebi.ac.uk/ena/browser/view/PRJEB48988 (WGS genebank collection) and https://www.ebi.ac.uk/ena/browser/view/SAMEA5374255 (Chinese Spring GBS). Details for the data used can be found in Material and Methods as well as in **Tables S1** and **S2**.

## Author contributions

JK designed the study and analyzed the data. All authors discussed the results. JK, SS, and BK drafted the manuscript. All authors contributed to the article and approved the submitted version.
