# Supplementary material for: Finding needles in a haystack: identification of inter-specific introgressions in wheat genebank collections using low-coverage sequencing data
Source: Front Plant Sci. 2023 Jun 6;14:1166854. doi: 10.3389/fpls.2023.1166854 (PMC10280009; doi:10.3389/fpls.2023.1166854)
Supplement: Supplementary file 1 [file DataSheet_1.pdf]

frontiers

Supplementary Material

Finding needles in a haystack: Identification of inter-specific introgressions in wheat genebank collections using low-coverage sequencing data

Corresponding author: Jens Keilwagen

Table S1: Introgressions identified in GBS collection. The table lists general information and the introgressions identified in each sample. The general information comprises the GBS ID, the IDs used (data used), the name of the sample, the number of introgressions detected by Schulthess et al. (2022), the ID of the single seed descent line (SSD-PDR), the cluster of duplicate detection, the ID of the corresponding WGS experiment, the panel of the sample, and the accession name if the sample is a genebank accession. The number of 1-Mb windows predicted as introgressions is given for the complete genome as well as for each individual chromosome.

Table S2: Introgressions identified in WGS collection. The table lists general information and the introgressions identified in each sample. The general information comprises the WGS ID, the IDs used (data used), the name of the sample, the number of introgressions detected by Schulthess et al. (2022), the ID of the single seed descent line (SSD-PDR), the cluster of duplicate detection, the historic period as given by Schulthess et al., the panel of the sample, and the accession name if the sample is a genebank accession. The number of 1-Mb windows predicted as introgressions is given for the complete genome as well as for each individual chromosome.

Table S3: Regions enriched with introgression predictions in elite cultivars using GBS data. The table lists regions with increased frequency of introgressions based on Fisher's exact test. The following information is given for each region: the chromosome, the start and end point in Mb, the peak, the LOD of the peak, the most frequent non-zero label of elite cultivars at the peak and the LOD for this specific label at the peak.

Table S4: Large introgressions in GBS and WGS collections. The table lists samples with at least one chromosome harboring a $\geq$ 300 Mb introgression. The following information is given for each sample: the experiment type, the sample ID, the name, the accession name if the sample is a genebank accession, the number of introgressions detected by Schulthess et al. (2022), the candidate chromosomes with at least one $\geq$ 300 Mb introgression, the detected rye DNA, and a comment field containing additional information.

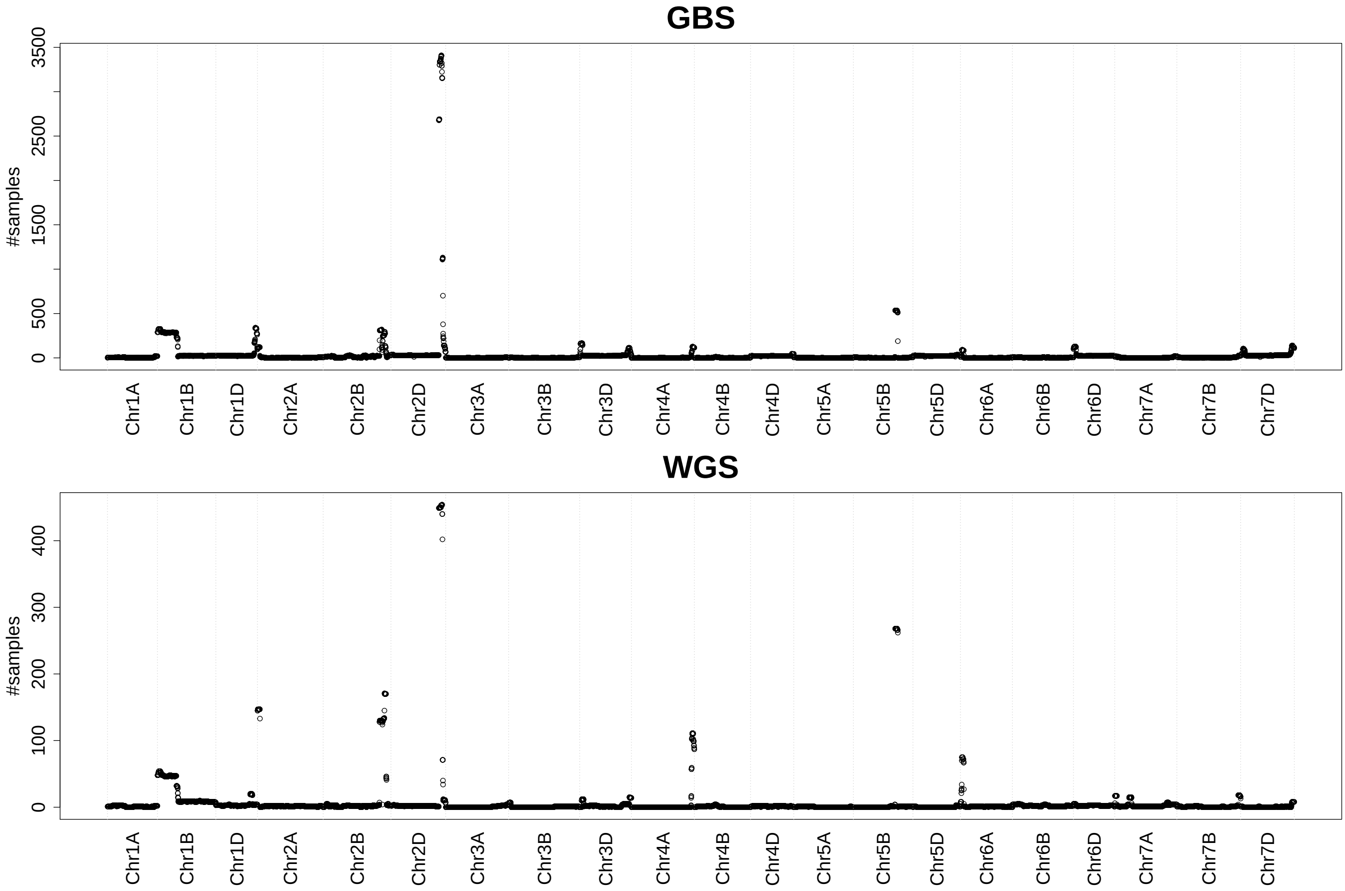

Figure S1. Chromosomal location of introgressions in GBS and WGS collections.

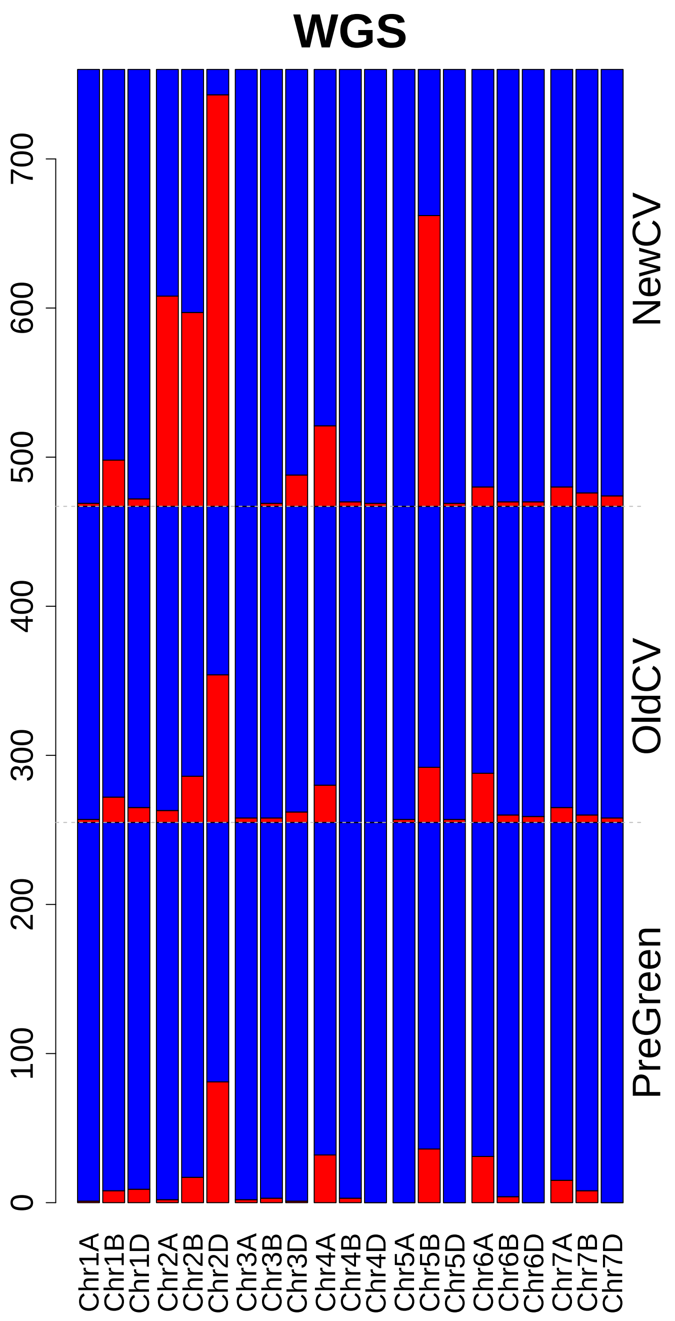

Figure S2. Number of samples with a predicted introgression in red, and number of samples without introgression in blue. Numbers for PRE-GREEN REVOLUTION PANEL (PreGreen, released before 1970), OLD CULTIVAR PANEL (OldCV, released between 1971 and 2000), and NEW CULTIVAR PANEL (NewCV, released after 2000) are stacked. Recently released cultivars show an enrichment of introgressions on chromosomes 2A, 2B, 2D, and 5B.

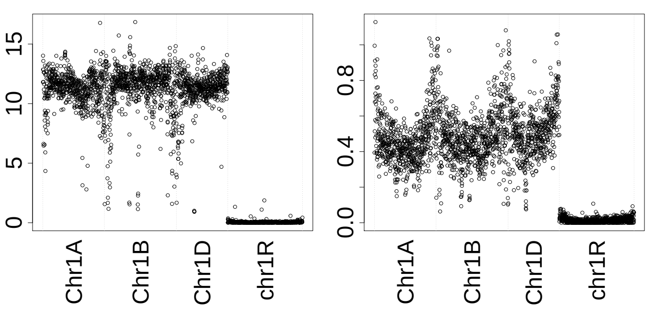

Figure S3a. Normal 1B: TRI 16401

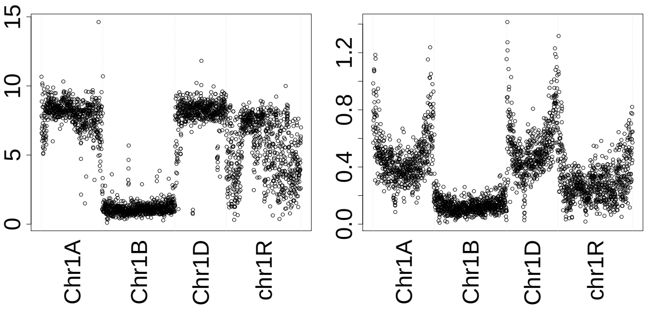

Figure S3b. 1R/1B: TRI 9323

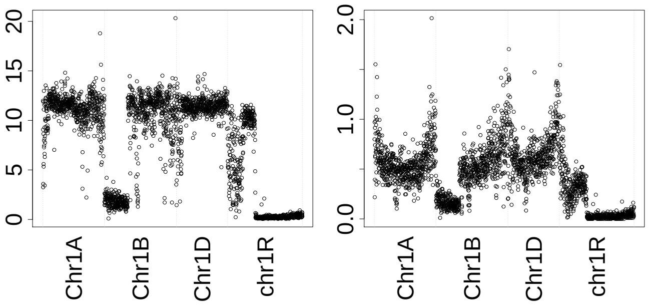

Figure S3c. T1RS.1BL: Gladiator

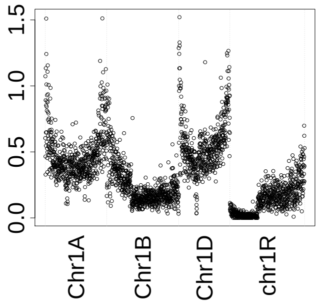

Figure S3d. T1BS.1RL: TRI 9467

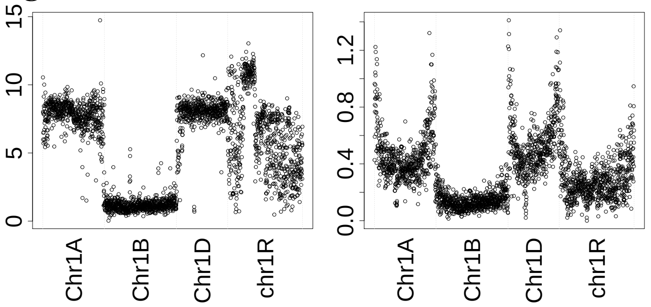

Figure S3e. Different SSD-PGR: TRI 3810 (Salzmünder 14/44)

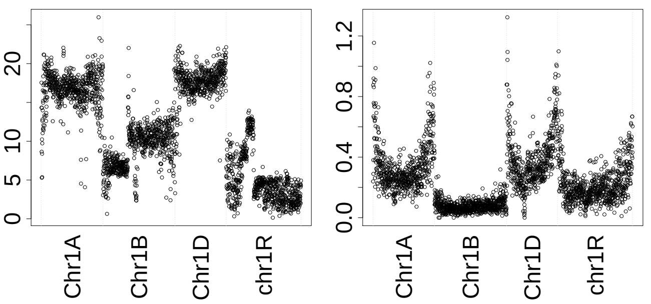

Figure S3f. Different accessions with similar name: Riebesel 47/51 (TRI 24963, TRI 8018)

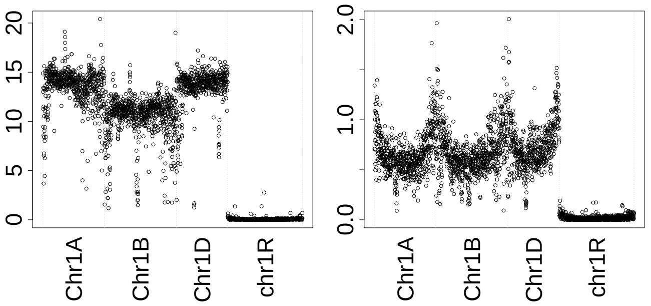

Figure S3g. non-rye substitution: LGMagirus

Figure S3. Overview of different introgressions on chromosome 1B. Dots show the percentage of covered bases in 1Mb windows. The scale on the y-axis depicts the percentage of covered bases and depends on the experiment (GBS or WGS) and the sequencing depth. The absolute values are not of interest, but rather the relation between them. The x-axis indicates the chromosomal location of chromosome 1A, 1B, 1D and 1R. a-d) Well-known cases of chromosome 1B depicted for WGS and GBS. e) two samples of TRI 3810 (Salzmünder 14/44) for different SSD-PGR. f) two samples of Riebesel 47/51 (TRI 24963 and TRI 8018). In e and f, the right panels depict 1R/1B genotypes using GBS data, while the left panels show strange introgression patterns of rye using WGS data. g) depicts WGS and GBS data for LGMagirus indicating a non-rye substitution based on WGS data.

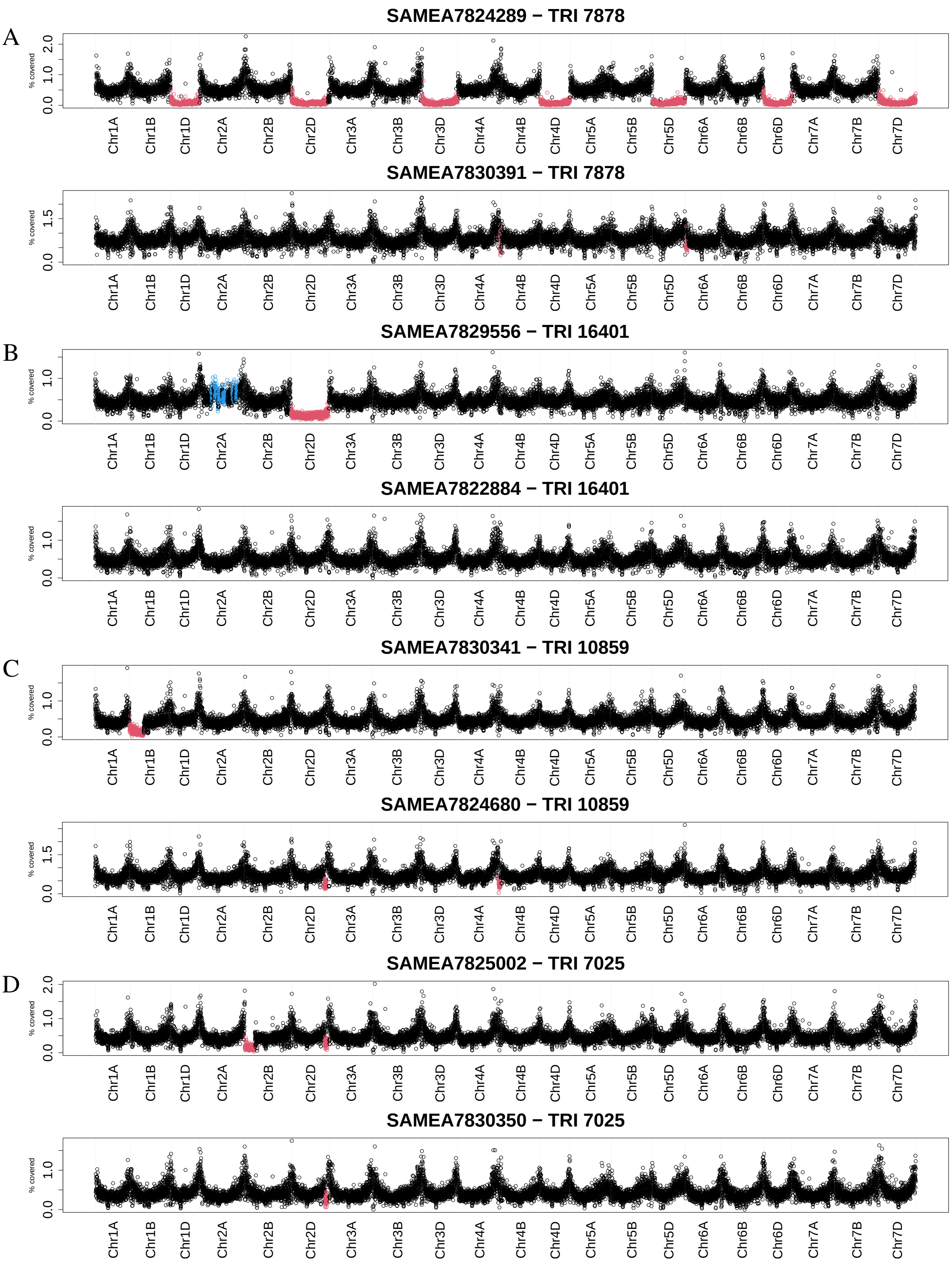

Figure S4. Heterogeneous genebank accessions from GBS collection. A) depicts a mixture of tetraploid and hexaploid wheat for TRI 7878. B) depicts a mixture of a wheat without introgression and a wheat with introgressions on chromosome 2A and 2D for TRI 16401. C) depicts the main difference on chromosome 1BS for the two subaccessions of TRI 10859. D) depicts the main difference on chromosome 2BS for the two subaccessions of TRI 7025.

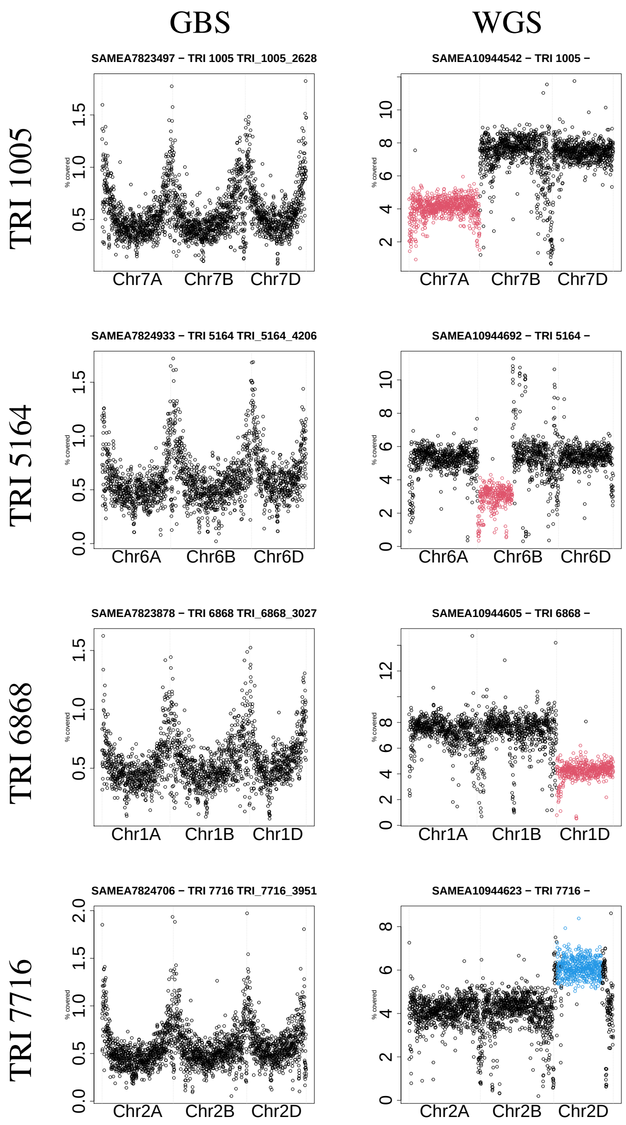

Figure S5. Accessions with unknown origin (SSD-PGR) and different introgression calls using GBS and WGS data. Dots show the percentage of bases covered in 1-Mb windows. X-axis indicates chromosomal location; scale on y-axis depicts the percentage of bases covered and depends on the experiment (GBS or WGS) and sequencing depth. Absolute values are not of interest, but rather the ratio between them. Consistent with introgression detection, black color indicates regions with expected percentage of bases covered. Red and blue colors indicate regions with an unexpectedly low and high percentage of bases covered, respectively.The left column depict predictions based on GBS, the right column depicts predictions based on WGS.

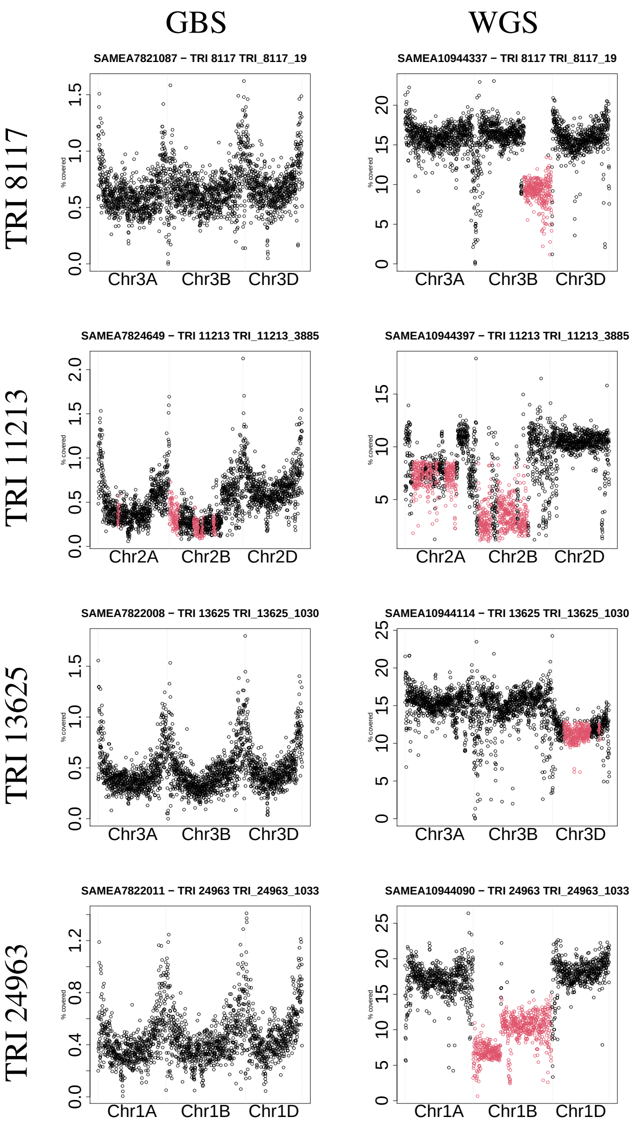

Figure S6. Accessions with same origin (SSD-PGR) and different introgression calls using GBS and WGS data. Dots show the percentage of bases covered in 1-Mb windows. X-axis indicates chromosomal location; scale on y-axis depicts the percentage of bases covered and depends on the experiment (GBS or WGS) and sequencing depth. Absolute values are not of interest, but rather the ratio between them. Consistent with introgression detection, black color indicates regions with expected percentage of bases covered. Red and blue colors indicate regions with an unexpectedly low and high percentage of bases covered, respectively.The left column depict predictions based on GBS, the right column depicts predictions based on WGS. The detection for TRI 11213 was difficult based on GBS data. TRI 24963 seems to be a mixture despite same origin (SSD-PGR) since rye substitutions were normally detected even with GBS.

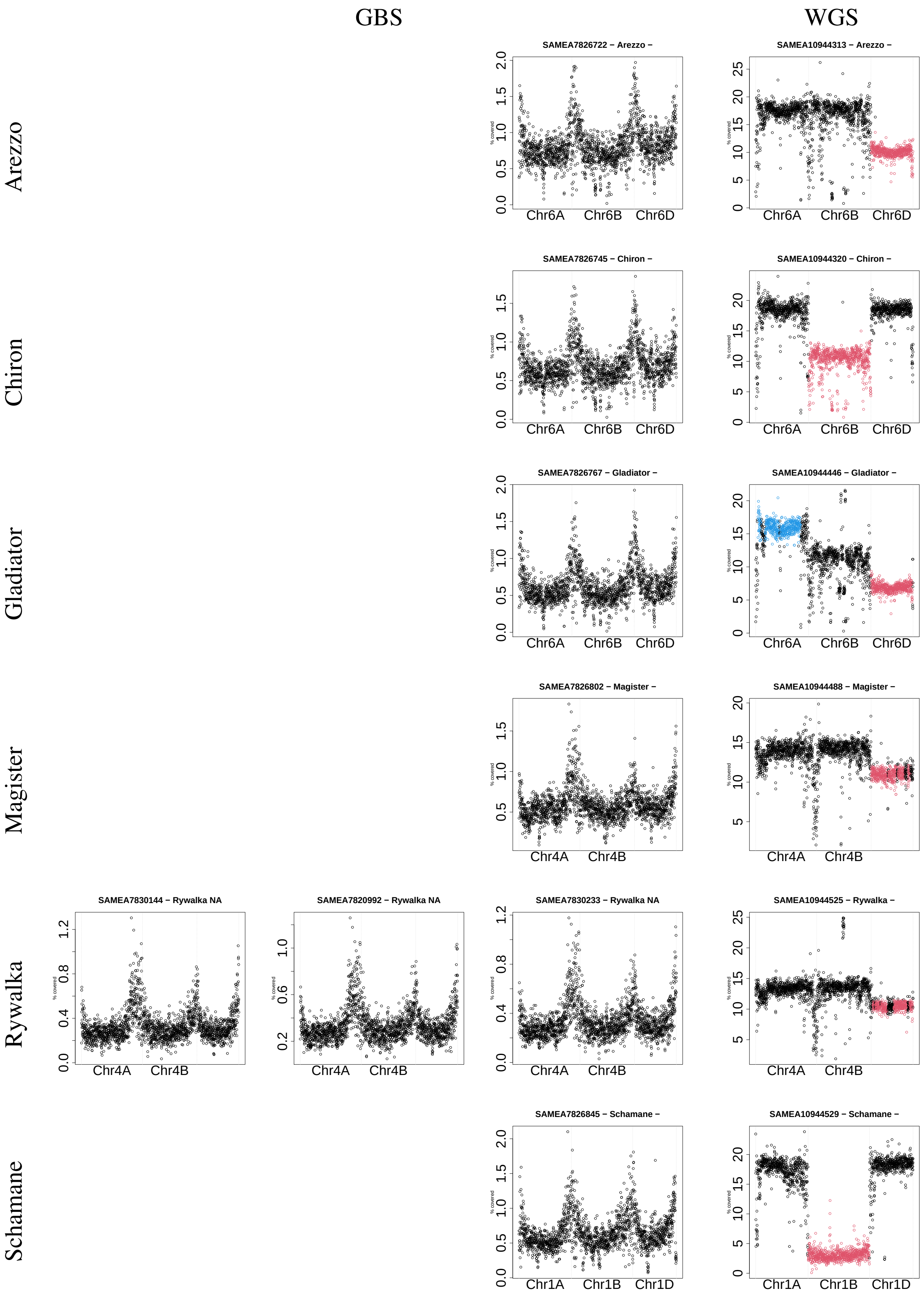

Figure S7. Cultivars with different introgression calls using GBS and WGS data. The left three columns depict predictions based on GBS, the right column depicts predictions based on WGS. Dots show the percentage of bases covered in 1-Mb windows. X-axis indicates chromosomal location; scale on y-axis depicts the percentage of bases covered and depends on the experiment (GBS or WGS) and sequencing depth. Absolute values are not of interest, but rather the ratio between them. Consistent with introgression detection, black color indicates regions with expected percentage of bases covered. Red and blue colors indicate regions with an unexpectedly low and high percentage of bases covered, respectively.

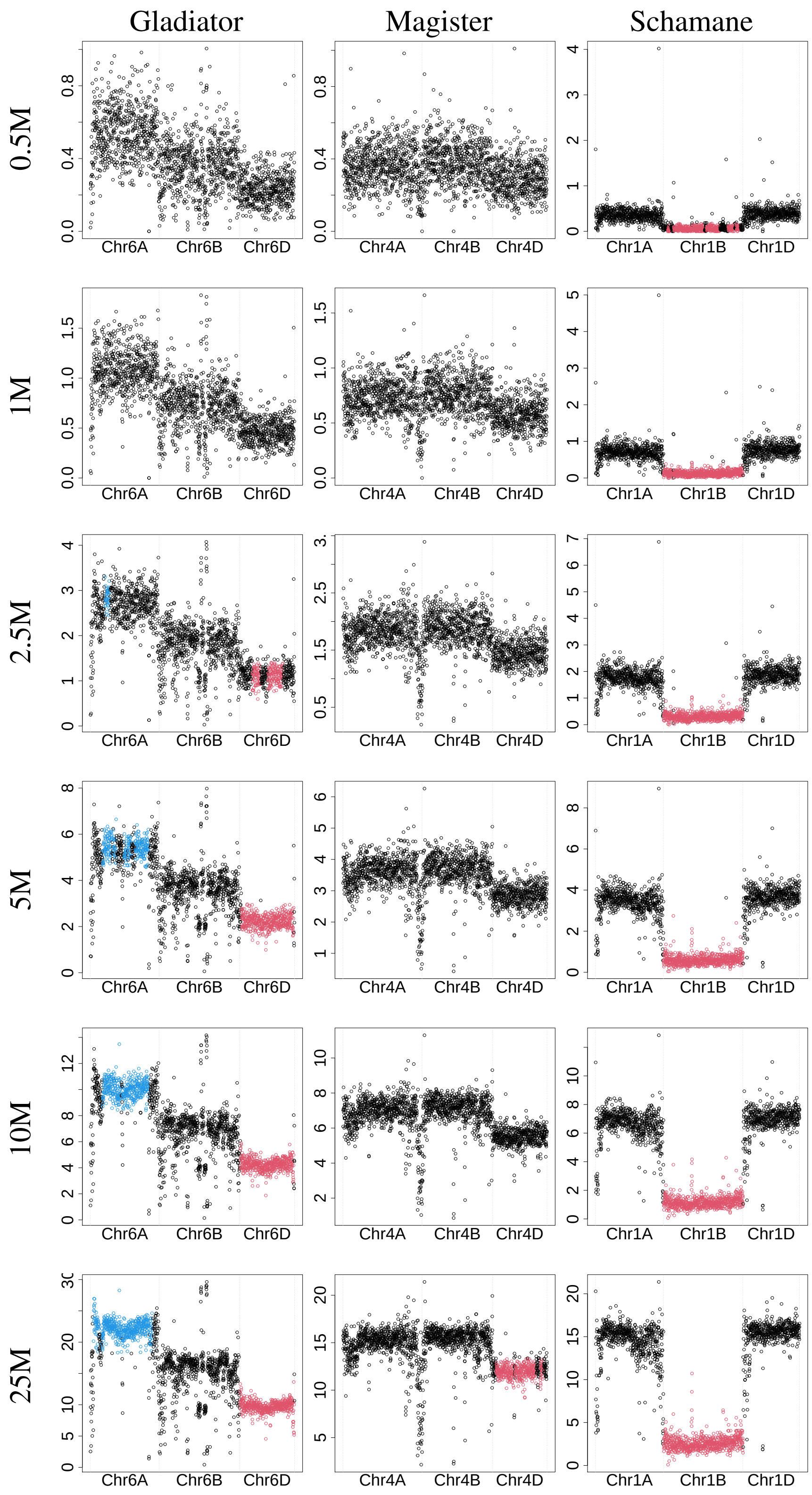

Figure S8. Profile of percentage covered bases for different number of WGS raw reads. Dots show the percentage of bases covered in 1-Mb windows. X-axis indicates chromosomal location; scale on y-axis depicts the percentage of bases covered and depends on the experiment (GBS or WGS) and sequencing depth. Absolute values are not of interest, but rather the ratio between them. Consistent with introgression detection, black color indicates regions with expected percentage of bases covered. Red and blue colors indicate regions with an unexpectedly low and high percentage of bases covered, respectively.
